# Severe sequelae in bilateral acute iris transillumination syndrome secondary to the use of oral moxifloxacin: a case report

**DOI:** 10.1186/s13256-021-03075-y

**Published:** 2021-09-19

**Authors:** Nicolás Rivera-Valdivia, Karla Arteaga-Rivera, Juliana Reyes-Guanes, Natalia Neira-Segura, Alejandra de-la-Torre

**Affiliations:** 1 Escuela Barraquer Research Group, Escuela Superior de Oftalmología - Instituto Barraquer de América, Avenida Calle 100 # 18A – 51, Bogotá, Colombia; 2 NeURos research group, Escuela de Medicina y Ciencias de la salud, Universidad del Rosario, Carrera 24 # 63 C 69, Bogotá, Colombia

**Keywords:** Drug-related side effects and adverse reactions, Moxifloxacin, Bilateral acute iris transillumination, Glaucoma, Sequelae

## Abstract

**Background:**

Moxifloxacin is a fourth-generation fluoroquinolone used as a second-line treatment for multiple bacterial infections. Uveitis has been described as an adverse effect related to this medication. Although several case reports have been published describing uveitis and bilateral acute iris transillumination syndrome related to moxifloxacin, we present a unique case of a patient with severe sequelae associated with bilateral acute iris transillumination syndrome secondary to the use of oral moxifloxacin.

**Case presentation:**

A 45-year-old Colombian hispanic female presented bilateral conjunctival hyperemia, decreased visual acuity, blurred vision, photophobia, and ocular pain after 15 days of treatment with systemic moxifloxacin for an upper tract respiratory infection. The patient presented unilateral anterior chamber pigment dispersion, mydriatic and nonreactive pupils, extensive iris transillumination defects, and secondary glaucoma. Blood and aqueous humor tests were negative for infectious and autoimmune diseases. Moxifloxacin-induced bilateral acute iris transillumination syndrome was diagnosed. Permanent sequelae such as ocular pain, photophobia, and focus difficulty secondary to severe bilateral iridian atrophy and inability of synkinetic reflex were left. Additionally, glaucoma was diagnosed, and Ahmed valve implantation was required.

**Conclusions:**

We should be aware of the possible association between moxifloxacin and bilateral acute iris transillumination syndrome. A detailed anamnesis, adequate examination, and laboratory tests are necessary to reach an early diagnosis and treatment to avoid unnecessary therapies. Larger studies should be carried out to understand the pathophysiology, diagnosis, management, and sequelae of the disease.

## Background

Fluoroquinolones are widely recognized and commonly prescribed antibiotics [1]. They inhibit the deoxyribonucleic acid (DNA) synthesis acting in DNA gyrase and topoisomerase IV, essential enzymes for bacterial replication and viability [2, 3]. Moxifloxacin is a fourth-generation fluoroquinolone, used as a second-line treatment for community-acquired pneumonia, bronchitis exacerbations, acute sinusitis, uncomplicated bacterial skin infections, abdominal abscess, and pelvic inflammatory disease, among others [1–5].

Several adverse effects have been related to these medications, including tendinitis, tendon rupture, peripheral neuropathy, diplopia, central nervous system alterations, arthralgias, myalgias, and QT interval prolongation, among others [5]. Bilateral acute iris transillumination (BAIT) was described as an adverse effect related to moxifloxacin for the first time in 2004 in a 77-year-old Spanish female [4].

After the first contact, moxifloxacin crosses the blood ocular barrier in non-inflamed eyes and reaches aqueous humor and vitreous within 4 hours [6]. All fluoroquinolones have shown vitreal penetration. Moxifloxacin has demonstrated a higher tissue affinity than other fluoroquinolones [7], which is why the main theory of ocular damage in BAIT proposes iris and ciliary body toxicity as the main cause [4, 6, 8].

Several case reports have been published describing BAIT related to moxifloxacin, most of them associated with iris transillumination and pigment dispersion syndrome [9, 10]. Nevertheless, here we present a unique case of a patient with permanent and severe sequelae associated with BAIT syndrome secondary to the use of oral moxifloxacin.

## Case report

We present a 45-year-old Colombian hispanic female patient with no prior relevant family or medical history, who reported a 2-year history of conjunctival hyperemia, decreased visual acuity, blurred vision, photophobia, and bilateral pain, associated with bilateral mydriasis and pigment dispersion. She was treated with topical prednisolone 1% without improvement. After observing a severe intraocular pressure (IOP) peak (35/37 mmHg), she was diagnosed with severe bilateral hypertensive anterior uveitis. Viral origin was suspected, and treatment with acyclovir was established with no improvement and hypertension persistence. After three months with mean IOPs of 28 mmHg OD and 30 mmHg OS, uveitic glaucoma was diagnosed, requiring surgical management with Ahmed valve implantation.

Infectious and autoimmune diseases were ruled out with blood tests, and a diagnosis of idiopathic uveitis was made. She was first treated with deflazacort and methotrexate, but no improvement was evidenced. Thus, treatment with an anti-TNF-α was established without resolution of the intraocular inflammation.

She was referred to our ocular immunology and uveitis consultation for a second opinion. During the anamnesis and the previous charts and prescriptions review, we noticed that she was treated with systemic moxifloxacin due to acute bronchitis 15 days before the onset of the ocular symptoms.

**Fig. 1:**
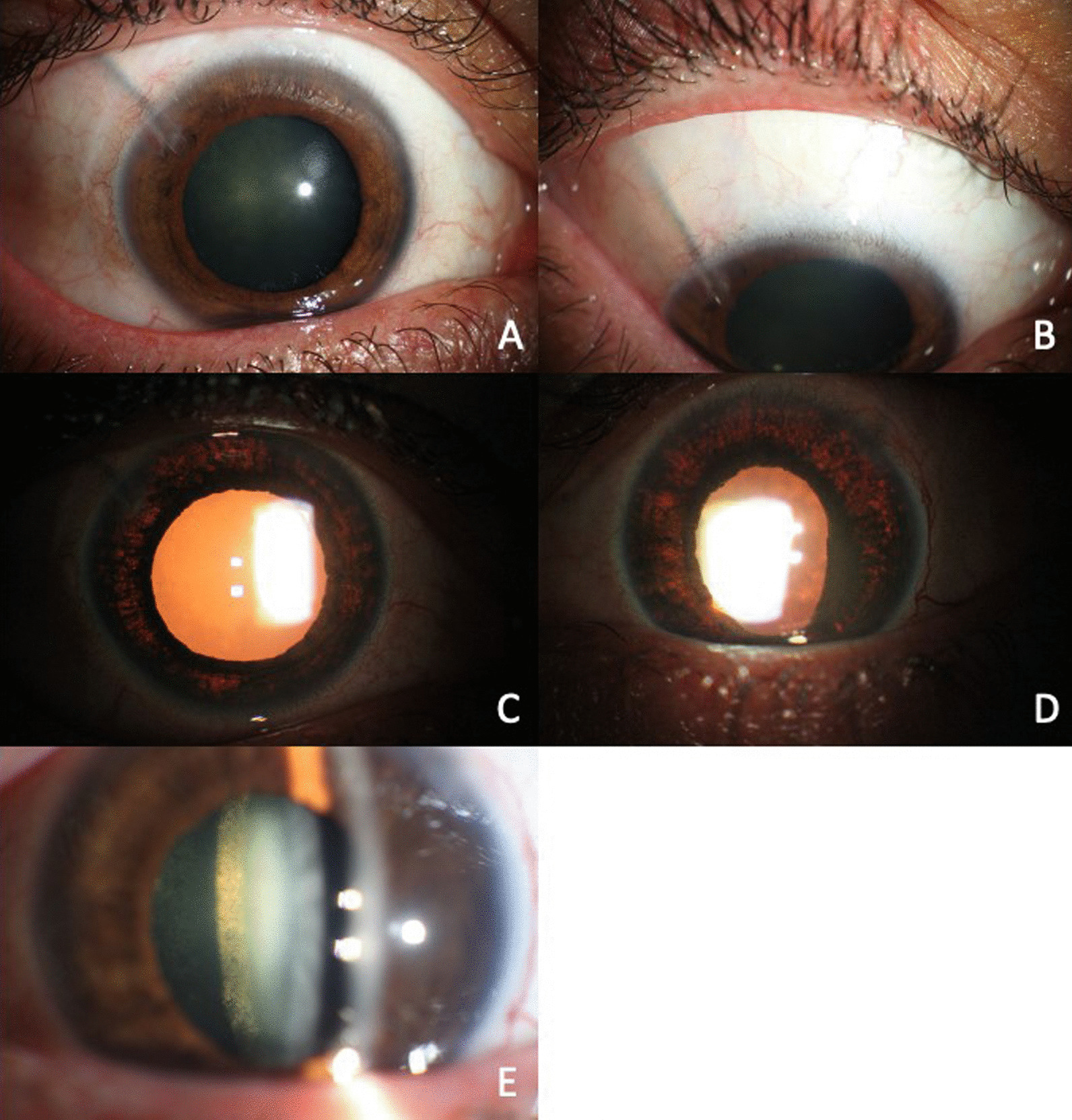
**A** and **B** Ahmed glaucoma valve. **C** and **D** Iridian atrophy with extensive transillumination defects in 360°. **E** Posterior subcapsular opacity

On ophthalmological examination, best-corrected visual acuity (BCVA) was 20/150 OD and 20/70 OS, and bilateral photophobia, anisocoria, and nonreactive right eye mydriasis were observed. On slit-lamp examination, positive findings were: OD—mild conjunctival hyperemia, Ahmed glaucoma valve (Fig. 1A and B), anterior chamber with no cells, mydriatic and nonreactive pupil, and iridian atrophy with extensive transillumination defects in 360° (Fig. 1C and D), OS—mild conjunctival hyperemia, Ahmed glaucoma valve, 3+ of pigment dispersion, mydriatic and nonreactive pupil, and iridian atrophy with extensive transillumination defects in 360° (Fig. 1C and D). Posterior subcapsular opacity was evidenced OU (Fig. 1E). IOP was 16/18 mmHg. A cup-to-disc ratio of 0.7 OD and 0.8 OS was observed, and the rest of the posterior segment examination was unremarkable OU.

Considering the patient presented common viral infection findings such as ocular hypertension, iridian atrophy defects, and anterior chamber inflammation, although viral uveitis is rarely bilateral, this diagnosis had to be discarded. Taking into account that blood laboratory tests were negative for infectious disease, polymerase chain reaction (PCR) in aqueous humor was requested OU. Results for viral origin, including cytomegalovirus, Epstein–Barr virus, herpes simplex virus (HSV) type 1 and 2, *Mycobacterium tuberculosis*, and toxoplasmosis, were negative.

Taking into account the mentioned history of acute bronchitis treated with oral moxifloxacin 15 days before the onset of ocular symptoms and the discard of an infectious and autoimmune etiology, moxifloxacin-induced BAIT syndrome was the final diagnosis. Permanent severe sequelae were found in this patient, including long-lasting eye pain, photophobia, and focus difficulty secondary to severe bilateral iridian atrophy and inability of synkinetic reflex. In the same way, Ahmed valve implantation was necessary for achieving intraocular pressure control.

The patient continued her treatment with topical antihypertensive drops (brinzolamide 1% + brimonidine tartrate 0.2% twice a day OD, and dorzolamide 2% + timolol 0.5% + brimonidine tartrate 0.2% twice a day OS) and topical lubricant drops. Treatment with anti-TNF-α was suspended after discarding autoimmune etiology. Resolution of pigment dispersion and stability of intraocular pressure were evidenced. Cataract surgery OU was performed. At the last follow-up, the patient was stable and presented a BCVA of 20/20 OD and 20/40 OS.

## Discussion

To the best of our knowledge, we present the first report of a patient with bilateral glaucoma development requiring Ahmed glaucoma valve implantation, the inability of synkinetic reflex, and iridian atrophy development as severe sequelae in BAIT syndrome secondary to the use of oral moxifloxacin. It is important to note that, due to these sequelae, the patient’s quality of life was severely affected.

BAIT has been relatively recently defined as an adverse effect of systemic moxifloxacin. The first case was reported in Spain in 2004; it described a 77-year-old patient who presented acute bilateral anterior “uveitis” with pigment dispersion after using moxifloxacin for pneumococcal pneumonia [10]. Since then, to the best of our knowledge, 17 cases have been described in the literature [7, 8, 10–17], but none of them has a clear pathophysiological mechanism. Currently, there is a lack of studies with high methodological quality regarding moxifloxacin toxicity in ocular tissue as the definite cause of BAIT. Thus, further investigation, such as laboratory-based experimental studies, is necessary to prove this relationship.

Moxifloxacin has been proven to form complexes with melanin, which may induce drug accumulation in melanin-rich tissues, such as the iris. This accumulation may produce toxic effects on melanin-containing cells [18]. Moreover, increased IOP presents in an early stage of BAIT syndrome and is usually refractory to treatment, causing glaucoma [19]. Both theories may contribute to the explanation of the severe sequelae presented by our patient.

BAIT syndrome is usually misdiagnosed as anterior uveitis, as during the acute, phase pigment dispersion might be confused with anterior chamber cellular Tyndall. Thus, it is often diagnosed and treated as anterior uveitis, as seen in our case. This is why gonioscopy in these patients is a great early diagnostic tool to observe initial trabeculum pigmentation [19, 20].

After systemic therapy with moxifloxacin, symptoms usually begin within 10–15 days. A detailed anamnesis and an adequate physical examination are important diagnostic tools to assess this issue [21, 22]. It usually presents with a bilateral course and stable visual acuity (≥ 20/40). The most common findings in BAIT secondary to systemic moxifloxacin are iris transillumination, followed by pigment dispersion, persistent mydriasis, conjunctival hyperemia, and photophobia. All of these signs were found in our patient. Additionally, as described in our patient, an elevated IOP, usually between 35 and 50 mmHg, is a typical finding as well, and it is usually attributed to pigment deposition in the trabecular meshwork [7, 10, 19]. We do not attribute ocular hypertension to steroid response, as an IOP rise was observed when tapered or suspended, which led us to think about an inflammatory component. In the same way, it is important to highlight that, although the IOPs in the 17 cases found in the literature and our case were within the range mentioned before, two of the cases presented a higher IOP than the one of our patient [7, 17]. Both of these patients required topical and oral antihypertensive treatment; one required peripheral iridectomy [7] and the other laser iridoplasty [17] with subsequent IOP decrease, oral treatment suspension, and topical treatment tapering. Still, none required additional device implantation or the use of biconjugated or triconjugated medications as our patient did.

Some findings of this drug-induced syndrome are frequently described in other ophthalmological entities. This is why it is important to note differential diagnoses such as viral uveitis, followed by Fuchs heterochromic iridocyclitis, pigmentary dispersion syndrome, and pseudoexfoliation syndrome [8, 15, 17, 23]. Aqueous humor PCR can be used as a diagnostic tool to rule out the infectious origin in these entities [21]. For example, we found that 4 of the 17 patients from the case reports and case series were studied with aqueous humor PCR for herpes viruses, but only one was positive for HSV [7, 8, 14, 17]. In our case, PCR was negative for herpes viruses, which reduced the possibility of having one of these diagnoses. In the same way, our patient did not present stellate keratic precipitates, heterochromia, blood vessels in the camerular angle, or iridian nodules as is common in Fuchs heterochromic iridocyclitis, but presented bilateral pain, red-eye, and photophobia, which are not common in this disease [24]. Additionally, although the patient presented transillumination defects, she did not present Krukenberg’s spindle, nor concave or bombe iris, common in pigmentary dispersion syndrome, but presented nonreactive mydriasis, which is not common in this illness. Moreover, this is usually presented in 30-year-old Black men, which is not the case in our patient [25]. Therefore, viral uveitis, Fuchs heterochromic iridocyclitis, and pigmentary dispersion syndrome were discarded.

From the 17 patients reported, all of them presented iridian atrophy as part of the BAIT syndrome. Nevertheless, no one presented Glaucoma as sequelae or Ahmed glaucoma valve requirement [7, 8, 10–17]. In the same way, only one patient presented posterior subcapsular opacity [16], as in our case. In Table 1, we present a comparison of clinical features between our case and other cases previously described in the literature.

**Table 1:** Comparison of clinical features with cases described in the literature

Author (year)	Number of cases	Time between first dose and onset of symptoms (days)	Empiric treatment	PCR for herpetic etiology study	Bilateral compromise	Anterior chamber cellularity	Transillumination defects	Pigment dispersion at gonioscopy	IOP OD; OS (mmHg)	BCVA OD; OS	Posterior subcapsular opacity	Glaucoma	Ahmed glaucoma valve
Rivera-Valdivia (present case)	1	14	Systemic antiviral and IMT	HSV (−) VZV (−) CMV (−) EBV (−) Mycobacteria (−) Toxoplasma (−)	+	+	+	+	30; 28	20/20; 20/40	+	+	+
Plaza-Ramos *et al.* (2018)	1	42	−	−	+	−	+	+	18; 18	20/20; 20/40	−	−	−
Rangel *et al.* (2017)	1	10	−	−	+	−	+	+	12; 12	20/20	−	−	−
Broens *et al.* (2016)	1	13	−	−	+	−	+	+	16; 16	20/20	−	−	−
Knape *et al.* (2013)	1	3	−	HSV (−) VZV (−) CMV (−)	+	−	+	+	35; 35	20/30; 20/40	−	ND	ND
Duncombe *et al.* (2013)	1	11	Systemic antiviral	HSV (−) VZV (−) CMV (−) EBV (−)	+	−	+	+	50; 28	20/25	−	−	−
Nascimento *et al.* (2013)	3	60–90	−	−	+ (2 pt)	+ (1 pt)	+	+ (1 pt)	14; 14 16; 16 12; 12	20/20; 20/20 20/20; 20/20 20/20; 20/30	−	−	−
Morshedi *et al.* (2012)	2	15–18	−	−	+	+	+	+	15; 26 15; 17	20/30; 20/40 20/20; 20/20	+ (1 pt)	−	−
Willermain *et al.* (2010)	1	15	−	Herpes viruses (−)	+	+	+	+	46; 46	20/200; 20/200	−	−	−
Wefers Bettink-Remeijer *et al.* (2009)	5	10–14	−	HSV (1 pt +)	+	+ (2 pt)	+	−	ND	ND	ND	ND	ND
Bringas Calvo *et al.* (2004)	1	10	−	−	+	+	+	+	9; 8	20/25; 20/30	−	−	−

*PCR* polymerase chain reaction, *IMT* immunomodulatory therapy, *IOP* intraocular pressure, *OD* right eye, *OS* left eye, *BCVA* best corrected visual acuity, *ND* not described, *pt* patient(s)

## Conclusion

We should be aware of the possible association between fluoroquinolones, especially moxifloxacin, and BAIT syndrome. It is important to consider this adverse reaction and the severe sequelae we described in our case. It is necessary to perform a detailed anamnesis with extensive patient history, adequate examination, and indicated laboratories to reach an early diagnosis and treatment. This could avoid unnecessary treatments, such as the ones used in our patient before the uveitis and ocular immunology specialist evaluation. Larger studies with high methodological quality should be carried out to understand the disease's pathophysiology, diagnosis, management, and sequelae.

## Data Availability

Data sharing not applicable to this article as no datasets were generated or analyzed during the current study.

## Abbreviations

IOP: Intraocular pressure
DNA: Deoxyribonucleic acid
OD: Right eye
OS: Left eye
OU: Both eyes
PCR: Polymerase chain reaction
HSV: Herpes simplex virus
TNF: Tumor necrosis factor
